# [Oxalylbis(aza­nedi­yl)]bis­{[amino­(2-pyrid­yl)methyl­ene]ammonium}

**DOI:** 10.1107/S1600536809009015

**Published:** 2009-03-19

**Authors:** Wen-Tao Bi, Nai-Liang Hu, Jian-Hong Bi

**Affiliations:** a School of Chemistry and Chemical Engineering, Anhui University, Hefei 230039, People’s Republic of China; b Department of Chemistry and Chemical Engineering, Hefei Teachers College, Hefei 230061, People’s Republic of China

## Abstract

The title compound, C_14_H_16_N_8_O_2_^2+^·2ClO_4_^−^, was prepared by reaction of bis­[amino­(2-pyrid­yl)methyl­ene]oxalohydrazide with perchloric acid. The mol­ecular symmetry is *C_i_* and thus the asymmetric unit comprises one half-mol­ecule. The dihedral angle between the aromatic ring and the plane of the oxamide group is 70.8 (3)°. The perchlorate anions and the cations are connected by inter­molecular N—H⋯O hydrogen bonds.

## Related literature

For background to the design and synthesis of polynuclear mol­ecule-based magnetic materials, see: Niel *et al.* (2008 ▶); Zhao *et al.* (2004 ▶); Xu *et al.* (2001 ▶, 2003 ▶).
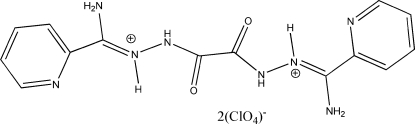


## Experimental

### 

#### Crystal data

C_14_H_16_N_8_O_2_^2+^·2ClO_4_^−^
*M* *_r_* = 527.25
Monoclinic, 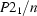

*a* = 5.0751 (11) Å
*b* = 13.725 (3) Å
*c* = 15.162 (3) Å
β = 98.605 (3)°
*V* = 1044.2 (4) Å^3^
*Z* = 2
Mo *K*α radiation
μ = 0.39 mm^−1^
*T* = 273 K
0.31 × 0.25 × 0.22 mm

#### Data collection

Bruker SMART CCD area-detector diffractometer
Absorption correction: multi-scan (*SADABS*; Bruker, 2000 ▶) *T*_min_ = 0.882, *T*_max_ = 0.914
5016 measured reflections
1824 independent reflections
1455 reflections with *I* > 2σ(*I*)
*R*_int_ = 0.078

#### Refinement

*R*[*F* ^2^ > 2σ(*F* ^2^)] = 0.045
*wR*(*F* ^2^) = 0.128
*S* = 1.09
1824 reflections
154 parameters
H-atom parameters constrained
Δρ_max_ = 0.33 e Å^−3^
Δρ_min_ = −0.47 e Å^−3^

### 

Data collection: *SMART* (Bruker, 2000 ▶); cell refinement: *SAINT* (Bruker, 2000 ▶); data reduction: *SAINT*; program(s) used to solve structure: *SHELXS97* (Sheldrick, 2008 ▶); program(s) used to refine structure: *SHELXL97* (Sheldrick, 2008 ▶); molecular graphics: *SHELXTL* (Sheldrick, 2008 ▶); software used to prepare material for publication: *SHELXTL*.

## Supplementary Material

Crystal structure: contains datablocks I, global. DOI: 10.1107/S1600536809009015/kp2209sup1.cif

Structure factors: contains datablocks I. DOI: 10.1107/S1600536809009015/kp2209Isup2.hkl

Additional supplementary materials:  crystallographic information; 3D view; checkCIF report

## Figures and Tables

**Table 1:** Hydrogen-bond geometry (Å, °)

*D*—H⋯*A*	*D*—H	H⋯*A*	*D*⋯*A*	*D*—H⋯*A*
N1—H1*A*⋯O4^i^	0.86	2.51	2.975 (3)	115
N1—H1*A*⋯O5^i^	0.86	2.58	3.404 (4)	162
N2—H2*A*⋯O3^ii^	0.86	2.21	2.970 (3)	147
N4—H4*A*⋯O1^iii^	0.86	2.23	2.974 (3)	145
N4—H4*B*⋯O2^iii^	0.86	2.05	2.820 (3)	148

Symmetry codes: (i) 
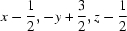
; (ii) 
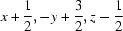
; (iii) 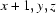.

**Fig. 1.**
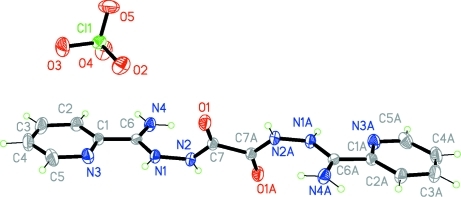
Molecular structure of the title compound, showing the 30% probability displacement ellipsoids and the atom-numbering [symmetry code: 1 - x,1 - y,1 - z].

**Fig. 2.**
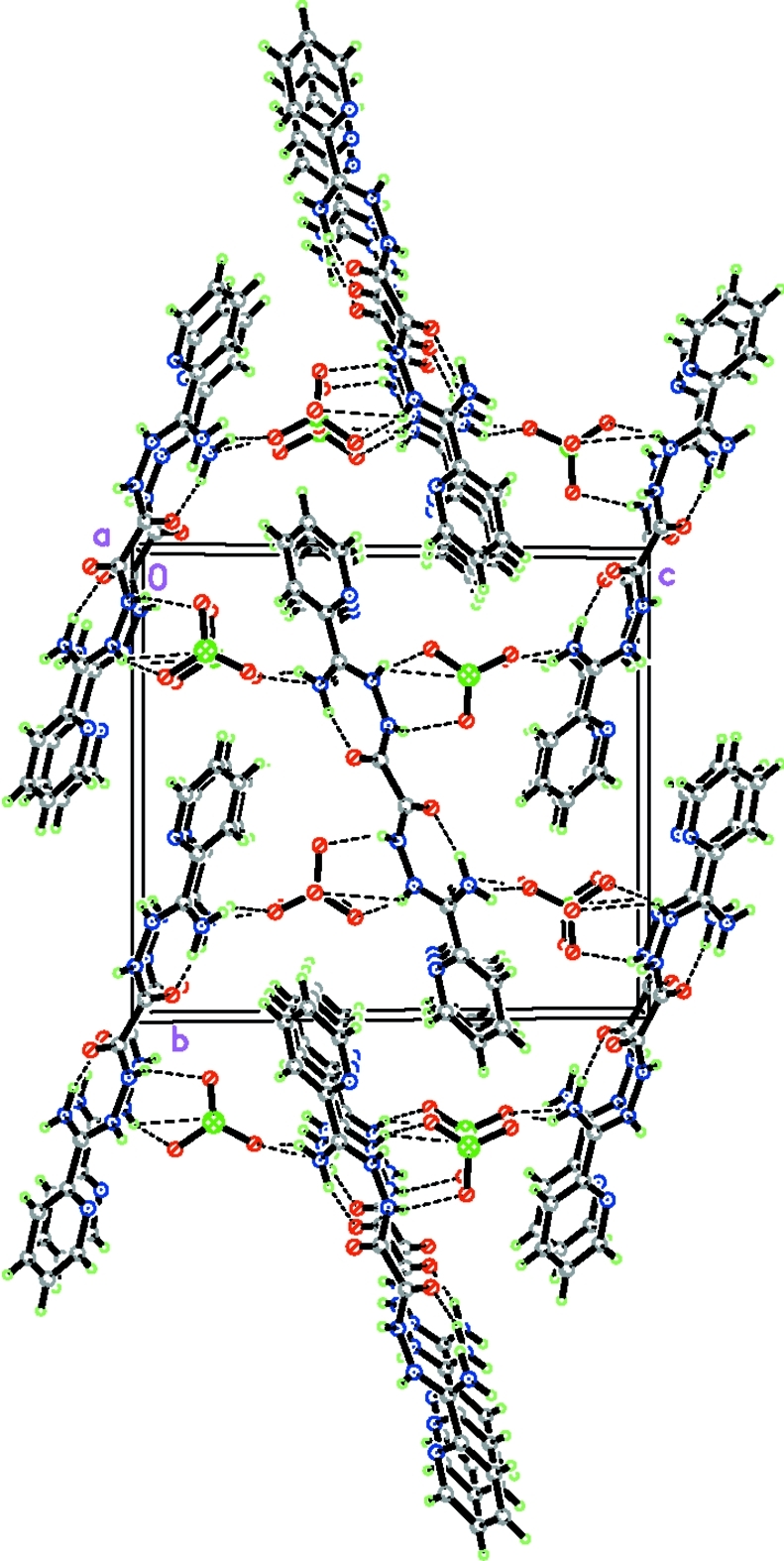
The crystal packing of the title compound generated by intermolecular hydrogen bonds.

## supplementary crystallographic information

### Comment

In recent years, researchers showed considerable interest in design and synthesis of polynuclear molecule-based magnetic materials, which were prepared by reactions of special organic molecules with transitional metals. (Niel *et al.*, 2008; Xu *et al.*, 2001; Xu *et al.*, 2003); Zhao *et al.*, 2004). Here we report a new compound, [(C_14_H_16_N_8_O_2_)(ClO_4_)_2_].

The asymmetric unit of the title compound comprises a half of the molecule (Fig. 1). In the structure of title compound, the dihedral angle between the aromatic ring and the plane of oxamide group is 70.8 °. Perchlorate anions and cations are connected by intermolecular N—H···O hydrogen bonds (Fig. 2, Table 1).

### Experimental

All solvents and chemicals were of analytical grade and were used without further purification. Ligand was prepared by similar procedure reported in the literature (Zhao *et al.*, 2004). For the synthesis of title compoud, a solution of ligand (0.1 mmol), HClO_4_(0.1 mmol) in 20 ml methanol was refluxed for 1 h, and then cooled to room temperature and filtered. Single crystals suitable for X-ray analysis were grown from the methanol solution by slow evaporation at room temperature in air. Anal. Calcd. for C_14_H_16_N_8_O_10_Cl_2_: C, 31.89; H, 3.06; N, 21.25. Found: C, 32.15; H, 3.18; N, 21.20.

### Refinement

All hydrogen atoms were geomemetrically positioned (C—H 0.93–0.97 Å, N–H 0.86 Å) and refined as riding, with *U*_iso_(H)=1.2 *U*_eq_ of the parent atom.

### Crystal data

**Table:**

C_14_H_16_N_8_O_2_^2+^·2ClO_4_^−^	*F*(000) = 540
*M**_r_* = 527.25	*D*_x_ = 1.677 Mg m^−^^3^
Monoclinic, *P*2_1_/*n*	Mo *K*α radiation, λ = 0.71073 Å
Hall symbol: -P 2yn	Cell parameters from 2046 reflections
*a* = 5.0751 (11) Å	θ = 2.7–26.2°
*b* = 13.725 (3) Å	µ = 0.39 mm^−^^1^
*c* = 15.162 (3) Å	*T* = 273 K
β = 98.605 (3)°	Block, colourless
*V* = 1044.2 (4) Å^3^	0.31 × 0.25 × 0.22 mm
*Z* = 2	

### Data collection

**Table:**

Bruker SMART CCD area-detector diffractometer	1824 independent reflections
Radiation source: fine-focus sealed tube	1455 reflections with *I* > 2σ(*I*)
graphite	*R*_int_ = 0.078
φ and ω scans	θ_max_ = 25.0°, θ_min_ = 2.0°
Absorption correction: multi-scan (*SADABS*; Bruker, 2000)	*h* = −6→5
*T*_min_ = 0.882, *T*_max_ = 0.914	*k* = −13→16
5016 measured reflections	*l* = −15→18

### Refinement

**Table:**

Refinement on *F*^2^	Primary atom site location: structure-invariant direct methods
Least-squares matrix: full	Secondary atom site location: difference Fourier map
*R*[*F*^2^ > 2σ(*F*^2^)] = 0.045	Hydrogen site location: inferred from neighbouring sites
*wR*(*F*^2^) = 0.128	H-atom parameters constrained
*S* = 1.09	*w* = 1/[σ^2^(*F*_o_^2^) + (0.076*P*)^2^] where *P* = (*F*_o_^2^ + 2*F*_c_^2^)/3
1824 reflections	(Δ/σ)_max_ = 0.001
154 parameters	Δρ_max_ = 0.33 e Å^−^^3^
0 restraints	Δρ_min_ = −0.47 e Å^−^^3^

### Special details

**Table:**

Experimental. The structure was solved by direct methods (Bruker, 2000) and successive difference Fourier syntheses.
Geometry. All e.s.d.'s (except the e.s.d. in the dihedral angle between two l.s. planes) are estimated using the full covariance matrix. The cell e.s.d.'s are taken into account individually in the estimation of e.s.d.'s in distances, angles and torsion angles; correlations between e.s.d.'s in cell parameters are only used when they are defined by crystal symmetry. An approximate (isotropic) treatment of cell e.s.d.'s is used for estimating e.s.d.'s involving l.s. planes.
Refinement. Refinement of *F*^2^ against ALL reflections. The weighted *R*-factor *wR* and goodness of fit *S* are based on *F*^2^, conventional *R*-factors *R* are based on *F*, with *F* set to zero for negative *F*^2^. The threshold expression of *F*^2^ > σ(*F*^2^) is used only for calculating *R*-factors(gt) *etc*. and is not relevant to the choice of reflections for refinement. *R*-factors based on *F*^2^ are statistically about twice as large as those based on *F*, and *R*- factors based on ALL data will be even larger.

### Fractional atomic coordinates and isotropic or equivalent isotropic displacement parameters (Å^2^)

**Table:**

	*x*	*y*	*z*	*U*_iso_*/*U*_eq_	
C1	0.7001 (5)	0.85329 (16)	0.62706 (14)	0.0356 (5)	
C2	0.8640 (5)	0.90729 (18)	0.68880 (16)	0.0487 (6)	
H2	1.0229	0.8819	0.7183	0.058*	
C3	0.7835 (7)	1.0013 (2)	0.70546 (19)	0.0654 (8)	
H3	0.8887	1.0402	0.7469	0.078*	
C4	0.5517 (8)	1.03584 (19)	0.6612 (2)	0.0679 (9)	
H4	0.4953	1.0987	0.6719	0.081*	
C5	0.4000 (6)	0.97677 (19)	0.6001 (2)	0.0604 (8)	
H5	0.2409	1.0013	0.5698	0.073*	
C6	0.7662 (4)	0.75221 (14)	0.60311 (13)	0.0326 (5)	
C7	0.4737 (4)	0.54717 (15)	0.52444 (14)	0.0352 (5)	
Cl1	0.54991 (12)	0.77141 (5)	0.85280 (3)	0.0450 (3)	
N1	0.5942 (4)	0.71127 (13)	0.53978 (12)	0.0370 (5)	
H1A	0.4530	0.7425	0.5175	0.044*	
N2	0.6387 (4)	0.61898 (12)	0.50903 (11)	0.0380 (5)	
H2A	0.7700	0.6079	0.4805	0.046*	
N3	0.4704 (4)	0.88577 (14)	0.58226 (14)	0.0486 (5)	
N4	0.9765 (4)	0.70721 (15)	0.64025 (12)	0.0445 (5)	
H4A	1.0082	0.6489	0.6240	0.053*	
H4B	1.0852	0.7355	0.6813	0.053*	
O1	0.3033 (4)	0.55207 (11)	0.57270 (12)	0.0506 (5)	
O2	0.4275 (4)	0.72826 (16)	0.77218 (13)	0.0710 (6)	
O3	0.4490 (5)	0.86811 (15)	0.85821 (15)	0.0793 (7)	
O4	0.8287 (4)	0.77292 (16)	0.85160 (15)	0.0730 (7)	
O5	0.4956 (6)	0.71688 (18)	0.92652 (16)	0.0973 (9)	

### Atomic displacement parameters (Å^2^)

**Table:**

	*U*^11^	*U*^22^	*U*^33^	*U*^12^	*U*^13^	*U*^23^
C1	0.0392 (13)	0.0310 (11)	0.0376 (11)	−0.0040 (9)	0.0085 (10)	−0.0016 (9)
C2	0.0529 (16)	0.0435 (14)	0.0490 (14)	−0.0095 (11)	0.0050 (11)	−0.0077 (11)
C3	0.085 (2)	0.0450 (16)	0.0675 (18)	−0.0155 (16)	0.0163 (17)	−0.0204 (14)
C4	0.096 (3)	0.0315 (15)	0.083 (2)	−0.0006 (15)	0.0361 (19)	−0.0080 (13)
C5	0.0628 (19)	0.0409 (15)	0.080 (2)	0.0110 (13)	0.0182 (15)	0.0084 (13)
C6	0.0362 (13)	0.0330 (11)	0.0287 (12)	−0.0032 (9)	0.0049 (9)	−0.0007 (9)
C7	0.0380 (13)	0.0308 (12)	0.0348 (12)	0.0014 (9)	−0.0012 (9)	−0.0029 (8)
Cl1	0.0409 (4)	0.0542 (4)	0.0375 (4)	0.0076 (3)	−0.0019 (3)	−0.0028 (2)
N1	0.0371 (11)	0.0292 (10)	0.0422 (10)	0.0005 (7)	−0.0027 (8)	−0.0062 (7)
N2	0.0419 (11)	0.0300 (10)	0.0419 (10)	−0.0024 (8)	0.0058 (8)	−0.0090 (8)
N3	0.0509 (13)	0.0354 (11)	0.0576 (12)	0.0061 (9)	0.0020 (10)	−0.0010 (9)
N4	0.0430 (12)	0.0399 (12)	0.0466 (12)	0.0047 (9)	−0.0069 (9)	−0.0093 (8)
O1	0.0580 (12)	0.0374 (9)	0.0614 (11)	−0.0018 (8)	0.0246 (9)	−0.0084 (8)
O2	0.0610 (14)	0.0874 (16)	0.0572 (12)	−0.0057 (10)	−0.0150 (9)	−0.0154 (10)
O3	0.0885 (16)	0.0567 (14)	0.0966 (15)	0.0244 (12)	0.0262 (13)	−0.0043 (11)
O4	0.0374 (12)	0.0868 (16)	0.0894 (16)	0.0060 (10)	−0.0079 (10)	−0.0147 (11)
O5	0.130 (2)	0.106 (2)	0.0648 (14)	0.0451 (16)	0.0439 (15)	0.0363 (13)

### Geometric parameters (Å, °)

**Table:**

C1—N3	1.335 (3)	C7—O1	1.215 (3)
C1—C2	1.373 (3)	C7—N2	1.336 (3)
C1—C6	1.485 (3)	C7—C7^i^	1.535 (4)
C2—C3	1.387 (4)	Cl1—O5	1.406 (2)
C2—H2	0.9300	Cl1—O2	1.415 (2)
C3—C4	1.351 (5)	Cl1—O4	1.418 (2)
C3—H3	0.9300	Cl1—O3	1.429 (2)
C4—C5	1.377 (5)	N1—N2	1.380 (2)
C4—H4	0.9300	N1—H1A	0.8600
C5—N3	1.338 (3)	N2—H2A	0.8600
C5—H5	0.9300	N4—H4A	0.8600
C6—N4	1.287 (3)	N4—H4B	0.8600
C6—N1	1.322 (3)		
			
N3—C1—C2	124.1 (2)	O1—C7—C7^i^	121.9 (2)
N3—C1—C6	113.56 (19)	N2—C7—C7^i^	112.3 (2)
C2—C1—C6	122.3 (2)	O5—Cl1—O2	110.53 (18)
C1—C2—C3	117.5 (3)	O5—Cl1—O4	109.43 (16)
C1—C2—H2	121.3	O2—Cl1—O4	107.75 (14)
C3—C2—H2	121.3	O5—Cl1—O3	109.51 (14)
C4—C3—C2	119.6 (3)	O2—Cl1—O3	108.86 (13)
C4—C3—H3	120.2	O4—Cl1—O3	110.74 (13)
C2—C3—H3	120.2	C6—N1—N2	120.78 (19)
C3—C4—C5	119.0 (3)	C6—N1—H1A	119.6
C3—C4—H4	120.5	N2—N1—H1A	119.6
C5—C4—H4	120.5	C7—N2—N1	118.65 (18)
N3—C5—C4	123.1 (3)	C7—N2—H2A	120.7
N3—C5—H5	118.4	N1—N2—H2A	120.7
C4—C5—H5	118.4	C1—N3—C5	116.6 (2)
N4—C6—N1	121.8 (2)	C6—N4—H4A	120.0
N4—C6—C1	123.0 (2)	C6—N4—H4B	120.0
N1—C6—C1	115.2 (2)	H4A—N4—H4B	120.0
O1—C7—N2	125.83 (19)		

Symmetry codes: (i) −*x*+1, −*y*+1, −*z*+1.

### Hydrogen-bond geometry (Å, °)

**Table:**

*D*—H···*A*	*D*—H	H···*A*	*D*···*A*	*D*—H···*A*
N1—H1A···O4^ii^	0.86	2.51	2.975 (3)	115
N1—H1A···O5^ii^	0.86	2.58	3.404 (4)	162
N2—H2A···O3^iii^	0.86	2.21	2.970 (3)	147
N4—H4A···O1^iv^	0.86	2.23	2.974 (3)	145
N4—H4B···O2^iv^	0.86	2.05	2.820 (3)	148

Symmetry codes: (ii) *x*−1/2, −*y*+3/2, *z*−1/2; (iii) *x*+1/2, −*y*+3/2, *z*−1/2; (iv) *x*+1, *y*, *z*.
